# Apoptosis-Inducing Effect of Ginsenoside Rg6 on Human Lymphocytoma JK Cells

**DOI:** 10.3390/molecules18078109

**Published:** 2013-07-09

**Authors:** Bin Chen, Xiao-Bin Jia

**Affiliations:** Key Laboratory of Chinese Medicine Delivery System of State Administration of Traditional Chinese Medicine, Jiangsu Provincial Academy of Chinese Medicine, 100 Shizi Road, Hongshan Street, Nanjing 210028,China; E-Mail: xiaobinjia_nj@126.com

**Keywords:** ginsenoside Rg6, steamed notoginseng, human lymphocytoma JK cell, cell apoptosis, mitochondrial dysfunction, Bcl-2 protein family

## Abstract

In this communication our aim was to study the JK cell growth inhibitory and apoptosis-inducing effects of ginsenoside Rg6 (GRg6) from steamed notoginseng on human lymphocytoma. The CCK-8 method was used to observe the anti-proliferative effect of GRg6 on human lymphocytoma JK cells. Flow cytometry was performed to analyze the influence of GRg6 on cell cycle. The Annexin-V FITC/PI double-staining method was used to detect the ratio of apoptotic cells. JC-1 staining was undertaken to observe the influence of GRg6 on intracellular mitochondrial membrane potential. Finally, western blots were conducted to detect the expression level of apoptosis-related Bax and the Bcl-2 proteins. The results suggested that GRg6 can inhibit the proliferation of human lymphocytoma JK cells. GRg6 blocks an *S* arrest in the cell cycle. With the increase in GRg6 concentration, the potential in the cell decreased in a dose dependent manner, and Bax protein expression gradually increased, whereas Bcl-2 protein expression gradually decreased. In conclusion, GRg6 can inhibit JK cell proliferation in human lymphocytoma and induce its apoptosis. The mechanism of action may be related to mitochondrial dysfunction and an increase of Bax expression and decrease of Bcl-2 expression caused by GRg6.

## 1. Introduction

The term lymphocytoma refers to the canceration of lymphocytes, *viz*. lymphoma. According to the ‘World Health Organization neoplasia pathological classification standard of the lymphatic system,’ the nearly 70 kinds of known types of pathological lymphoma can generally be divided into two categories: Hodgkin lymphoma and non-Hodgkin lymphoma. In China, Hodgkin lymphoma accounts for 9% to 10% of lymphoma cases, and is a malignant tumor that has a relatively good curative effect. Non-Hodgkin lymphoma accounts for about 90% and has shown a yearly increasing incidence in the last decade. 

**Figure 1:**
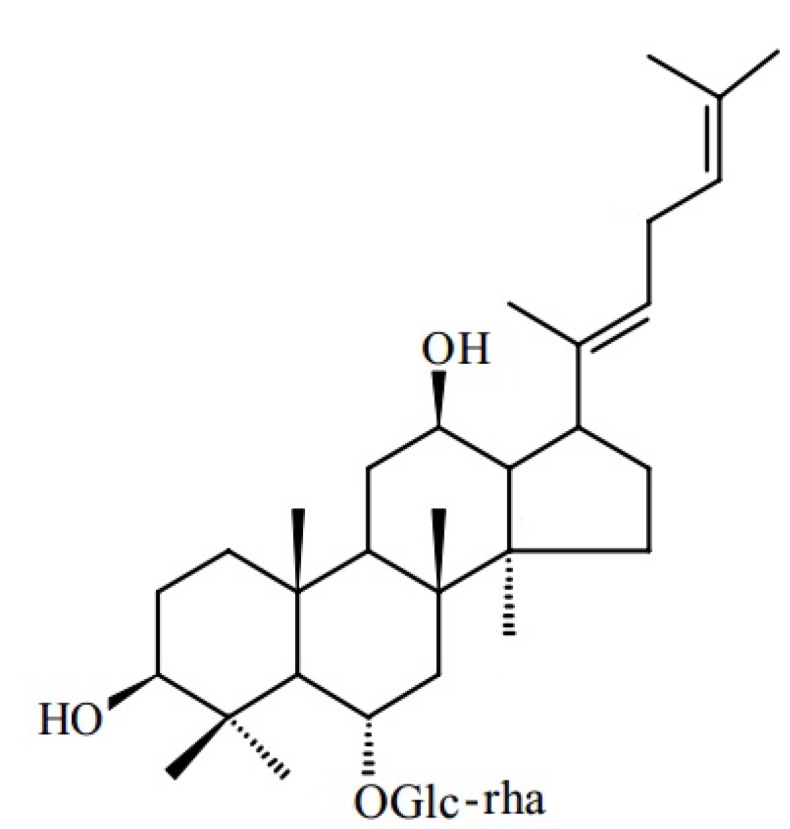
Chemical structure of GRg6.

Currently, chemotherapy is the main treatment for lymphoma, but the side effects are a cause of concern. Therefore, searching for and developing a treatment for lymphoma using Chinese herbal medicine has elicited extensive interest. Notoginseng is a common traditional Chinese herbal medicine that can promote blood circulation, remove blood stasis, and relieve swelling and pain. Notoginseng is mainly found distributed in Yunnan and Guangxi. The raw product functions in hemostasis, blood stasis dispersion, detumescence, and pain relief. Its processed products function in blood replenishment, immunization boosting, tumor inhibition, and muscular strengthening. Shi Sun [1] and other researchers found that the saponin composition of notoginseng changes after steaming. They studied and measured the composition and antitumor activity of notoginsenoside processed with different steaming times and temperatures. The results indicated that steamed notoginseng extracts could significantly inhibit the activity of SW-480 cell proliferation in human colorectal cancer. Compared with the ginseng ginsenosides Rg1 and Rb1, ginsenoside Rg3 could significantly inhibit the proliferation. Toh [2] and other researchers found that ingredients such as ginsenoside Rk3, Rh2, Rg3, and Rk1 abundant in steamed notoginseng can inhibit SNU449, SNU182, and HepG2 hepatocellular carcinoma cell proliferation. GRg6 is the component isolated from notoginseng by steaming (Figure 1). A previous report [3] discussed the function of GRg6 in inhibiting platelet aggregation, but no report is available regarding the effect of GRg6 on human lymphocytoma JK cells. Moreover, its mechanism remains unclear.

In this study, the effect of GRg6 on the *in vitro* growth and apoptosis of the JK cell of human lymphocytoma was reported and its mechanism of action was discussed. The information presented will provide a scientific basis for developing GRg6 as a chemical prophylaxis and treatment for human lymphocytoma.

## 2. Results and Discussion

### 2.1. Influence of Different Concentrations of GRg6 on Cell Proliferation

**Figure 2:**
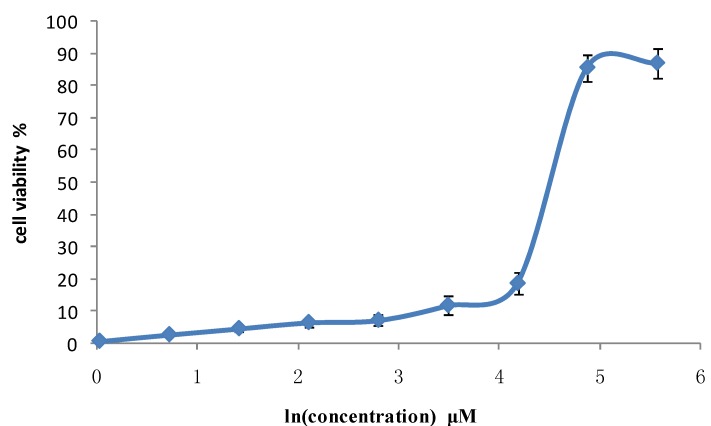
Anti-proliferative effects of GRg6 on human lymphocytoma JK cell.

CCK-8 method showed that after GRg6 was used, several groups with different concentrations obviously inhibited JK cell proliferation in human lymphocytoma, with evident dose dependency. Based on IC_50_, the median inhibitory concentration of GRg6 was 83.08 μM (Table 1) (Figure 2).

**Table 1:** Cell proliferation detecting using CCK-8 method.

Group (μM)	GRg6		
	OD ± SD	IR (%)	IC_50_
Negative	1.181 ± 0.013	—	83.08 μM
Positive	0.150 ± 0.003	87.27	
1.02	1.171 ± 0.015	0.82	
2.04	1.147 ± 0.018	2.85	
4.08	1.125 ± 0.011	4.74	
8.15	1.102 ± 0.021	6.63	
16.31	1.094 ± 0.017	7.34	
32.62	1.040 ± 0.014	11.94	
65.23	0.959 ± 0.006	18.75	
130.46	0.169 ± 0.002	85.71	
260.93	0.152 ± 0.002	87.10	

### 2.2. Hochest33258 Fluorescence Staining to Detect Cell Apoptosis

**Figure 3:**
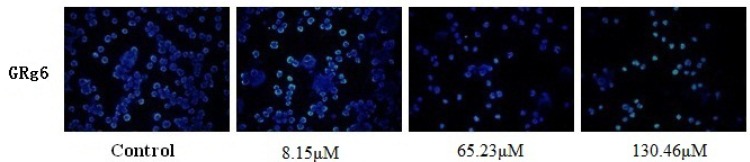
Hochest 33258 fluorescence staining detection of human lymphocytoma JK cell apoptosis treated with GRg6.

The CKK-8 experimental test showed that GRg6 has certain toxic effects on JK cells. Low, medium, and high concentrations were selected for immunofluorescence and flow detection in the cell apoptosis experiment. Results showed that with the increase of GRg6 concentration, apoptosis obviously increases and the dose dependency is significant (Figure 3).

### 2.3. Annexin-V FITC/PI Double-staining Method to Detect Cell Apoptosis

**Figure 4:**
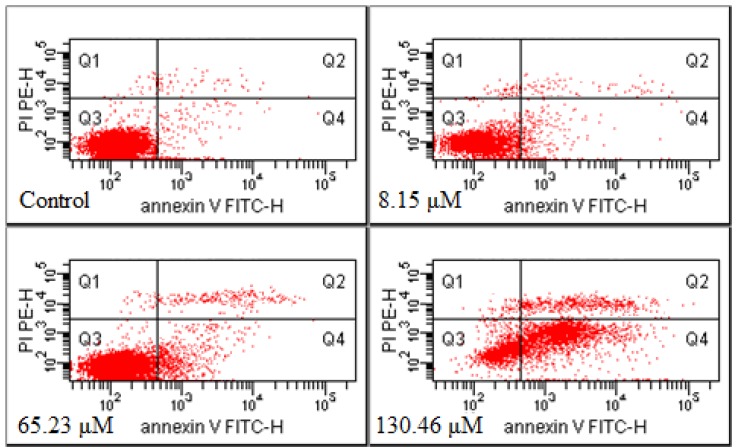
Measurement of apoptotic rates by Annexin V FITC/PI analysis.

After the Annexin-V FITC/PI double-staining method was adopted to treat the cells of the control group and the GRg6-treated group, three groups of cell population were presented in the flow cytometry: normal cells (Annexin V−/PI−), viable apoptotic cells (Annexin V+/PI−), and end-stage apoptotic and necrotic cells (Annexin V+/PI+). Up to 65.23 and 130.46 μM GRg6 induced cell apoptosis after treating the human lymphocytoma JK cells for 72 h. The Q1(%) were 1.05% and 1.25%, and the Q3(%) were 77.55% and 24.05%. The respective apoptosis rates [Q2(%) + Q4(%)] were 21.35% and 74.75%, showing certain dose dependency (Figure 4).

### 2.4. PI Single-Staining Method to Detect the Cell Cycle

**Figure 5:**
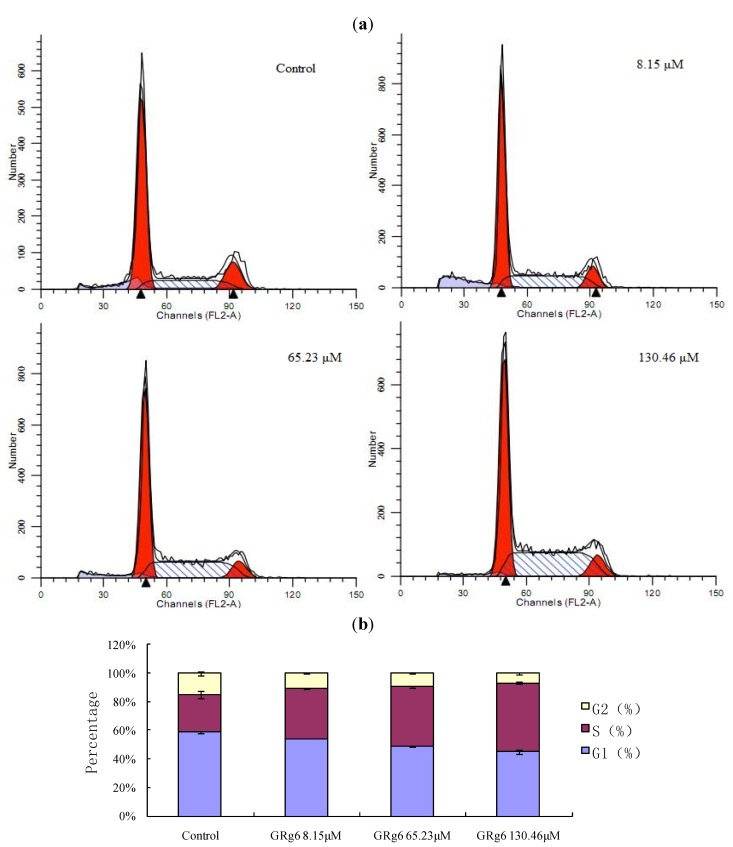
(**a**) Measurement of the cell cycle by PI single-staining analysis. (**b**) Comparison of the cell cycle with different concentrations of GRg6.

According to flow cycle detection by the PI single-staining method, GRg6 blocked *S* arrest in the cell cycle, and this effect becomes more obvious with the increase of medicine concentration Figure 5a,b.

### 2.5. JC-1 Staining Method to Detect Mitochondrial Membrane Potential

**Figure 6:**
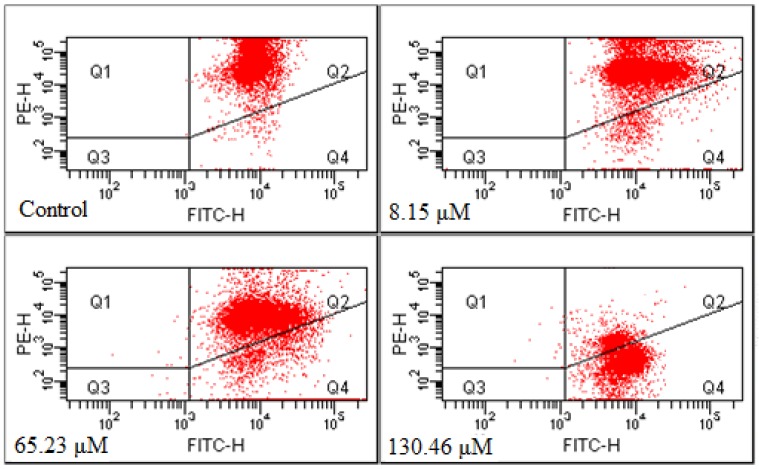
Measurement of mitochondrial membrane potential by JC-1 analysis.

After 20 min, the GRg6 depolarization cell ratio increased from 1.55% in the control group to 6.30%, 18.85%, and 82.85%, which indicates that the mitochondrial membrane potential of human lymphocytoma JK cells significantly decreased and exhibited a certain dose dependency (Figure 6).

### 2.6. Western Blot to Detect Protein Expression

**Figure 7:**
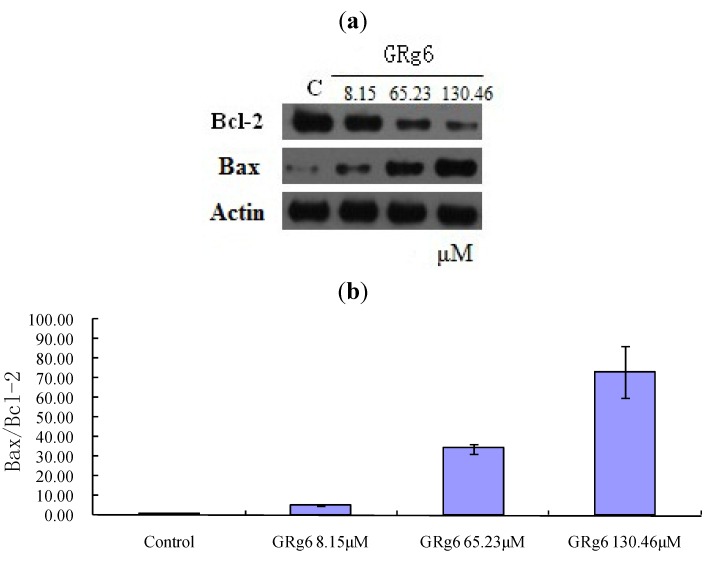
(**a**) Effects of GRg6 on Bax and Bcl-2 expression related to apoptosis. (**b**) Effects of GRg6 with different concentrations on Expression of Bax/Bcl-2 in human lymphocytoma JK cell.

According to the western blot results, compared with the control group, Bax protein expression was gradually enhanced and Bcl-2 expression was gradually weakened with the increase of the medicine concentration after the action of GRg6 on the human lymphocytoma JK cell Figure 7a,b.

### 2.7. Discussion

Several studies have shown that triterpenoid saponin compounds extracted and separated from a variety of ginseng, red ginseng, raw notoginseng, and cooked notoginseng have good antitumor effects *in vivo* and *in vitro* [4,5]. Triterpenoid saponin compounds in steamed notoginseng, especially GRg6, have the same cytotoxic effect. This experimental study found that GRg6 can generate obvious anti-proliferative effects when applied to JK cells *in vitro*. According to the cell cycle analysis, GRg6 blocks an *S* arrest in the cell cycle. Based on the detection of apoptosis rate by Annexin-V FITC/PI double staining, the apoptotic cell ratio significantly increased after the action of GRg6. Therefore, GRg6 can induce JK cell apoptosis.

Many mechanisms can cause cell apoptosis, including the death receptor-mediated signal pathway, mitochondria mediation, traditional signal mediation, T cell receptor signal transduction and endoplasmic reticulum mediation, among which the mitochondria function as the main switches in apoptosis. The early sign of mitochondrial dysfunction is the decline of the potential difference between the inner and the outer mitochondrial membrane, which can cause a series of biochemical reactions, such as increased mitochondrial membrane permeability, release of cytochrome C, activation of the caspase family proteins and cell apoptotic cascade reaction [6].

According to the JC-1 staining results, after the action of GRg6, the intracellular mitochondrial membrane potential noticeably declined. According to the western blot results, GRg6 can control Bax and Bcl-2 proteins in the Bcl-2 protein family of the mitochondrial membrane, affect the ion channel switch, increase the internal flow of Ca^2+^, generate reactive oxygen species, promote the release of cytochrome C and other apoptotic factors, and finally cause cell apoptosis. The experiments on induction of cytochrome C release from mitochondria to cytosol in human lymphocytoma JK cells and inhibiting activation of the caspase family proteins is still underway and the results need to be evaluated.

According to the result of the detection of cell cycle, we speculate that the mechanisms of action of GRg6 on the cell cycle and cell apoptosis could be connected with the cyclins or cell cycle related proteins such as p21, p53, *etc.* The study of cyclins or cell cycle-related proteins including p21, p53, *etc*. is still undergoing and the results will be reported in due course once completed. 

## 3. Experimental

### 3.1. Materials

#### 3.1.1. Cell Strain

Human lymphocytoma JK cells were provided by the Nanjing Keygen Biotech Development Company (Nanjing, China), and 90%RPMI-1640+10%CS was used as the complete medium. The cells were cultured at 37 °C in a 5% CO_2_ incubator with saturated humidity.

#### 3.1.2. Medicine and Groups

Experimental medicine: GRg6 with purity above 98% according to high-performance liquid chromatography detection was prepared at our lab. The concentration of the stock solution prepared using sterile water was 10 mg/mL. The solution was stored at 4 °C for subsequent use. Grouping according to the CCK-8 experimental results was: Control, 8.15 μM GRg6, 65.23 μM GRg6, and 130.46 μM GRg6.

#### 3.1.3. Main Reagents and Consumables

Cell culture bottles (FALCON 353014, San Jose, CA, USA), RPMI-1640 (GIBCO 31800-105, St. Louis, MO, USA), CS (Hangzhou Sijiqing Biological Engineering Materials Co., Ltd., Hangzhou, China), 96-well cell culture plate (Corning Incorporated 3599, St. Louis, MO, USA), 6-well cell culture plate (Corning Incorporated 3516), paraformaldehyde (Nanjing Chemical Reagent Co., Ltd. 30525-89-4, Nanjing, China), Hochest33258 fluorescence staining kits (Beyotime Institute of Biotechnology C1017, Shanghai, China), EP pipes (AXYGEN, MCT-150-C, St. Louis, MO, USA), PVDF (PALL, 65421, St. Louis, MO, USA), and X-ray film (Shanghai ShenBei Photosensitive, Shanghai, China). Penicillin and streptomycin mixture (KGY002), PBS (KGB500), CCK-8 (KGA317), Annexin V-FITC apoptosis detection kit (KGA105), cell cycle detection kit (KGA511), mitochondrial membrane potential detection kit (JC method, KGA601), holoprotein extraction kit (KGP250), Bradford protein content detection kit (KGA801), 5×SDS–PAGE loading buffer (KGP101), SDS–PAGE gel preparation kit (KGP113), prestained protein molecular weight (KGP441), 10×Tris-glycine protein electrophoresis buffer (KGP103), Coomassie brilliant blue staining protein detection kit (KGP1001), 10× electrotransfer buffer (KGP102), Ponceau staining fluid (KGP105), 10× WB detergents (KGP109), WB confining liquid (KGP108), WB primary antibody dilution buffer (KGP106), WB secondary antibody dilution buffer (KGP107), WB antibody clearing buffer (KGP110), ECL detection kit (KGP1123), internal control primary antibody (β-Actin) (KGAA001), secondary antibody (sheep anti-mice/rabbit IgG-HRP), and visualizing and fixing kit (KGP11) were all purchased from Nanjing Keygen Biotech Development Company of China.

### 3.2. Methods

#### 3.2.1. Cell Culture

Human lymphocytoma JK cells were cultured in an RPMI-1640 nutrient solution containing 10% calf serum under 37 °C saturated humidity and 5% CO_2_, and then passaged for 2 to 3 days at a time.

#### 3.2.2. CCK-8 Method to Measure Cell Proliferation

The cells were counted and then a cell suspension with a concentration of 5 × 10^4^ cell/mL was prepared. About 100 µL cell suspension was added into the 96-well cell culture plate (5 × 10^3^ cells for each well), which was placed in the 5% CO_2_ incubator for 24 h at 37 °C. The medicine was diluted to the required concentration with a complete medium, and then 100 μL corresponding medicated medium was added into each well. The negative control group and positive control group were established. The 96-well cell culture plate was placed in the 5% CO_2_ incubator for 72 h at 37 °C. Subsequently, CCK-8 staining on the 96-well plate was performed by adding 10 μL CCK-8 in each well, which was cultured for 3 h in the incubator and then gently blended for 10 min by using a shaker. The absorbance of each well (OD) was measured with a microplate reader at 450 nm wavelength to calculate the inhibition rate. The cell proliferation experiment was duplicated using the MTT method. Evaluation of the IC_50_ using the CCK-8 method has been validated.

#### 3.2.3. Hochest33258 Fluorescence Staining

The cells were fixed with paraformaldehyde or 4% formaldehyde, immersed in PBS for 5 min, and then washed three times. An appropriate amount of Hoechst 33258 dyeing liquid was added, which fully covered the cells. The solution was set aside for 10 min at room temperature, immersed in PBS for 5 min, washed three times, mounted with anti-fluorescence quenching liquid seal pieces, and then observed under a fluorescence microscope.

#### 3.2.4. Annexin-V FITC/PI Double Staining Method to Detect Cell Apoptosis

The cells were dissociated and inoculated at the logarithmic phase into a six-well plate. The next day, the corresponding medicated medium was added according to the group setting. The negative control group was established. The cells were collected 72 h after the drug action. The cells were washed with PBS twice (centrifuged at 2,000 rpm for 5 min) to collect 5 × 10^5^ cells. Then, 500 μL binding buffer suspension was added to the cells. Afterward, 5 μL PI was added and evenly blended after 5 μL Annexin V-FITC was added. The solution was reacted for 5 min to 15 min at room temperature without light. Finally, cell apoptosis was detected with flow cytometry (Ex = 488 nm; Em = 530 nm).

#### 3.2.5. PI Single-Staining Method to Detect the Cell Cycle

The cells were dissociated and inoculated at the logarithmic phase into a six-well plate. The next day, the cells were dissociated, collected, and washed with PBS (centrifuged at 2,000 rpm for 5 min) to collect and adjust the cell concentration to 1 × 10^6^/mL. The prepared single-cell suspension was fixed with 70% volume fraction ethanol for 2 h and stored at 4 °C. The stationary liquid was washed with PBS before staining. A total of 100 μL RNase A was added, and then the solution was placed in a water bath for 30 min at 37 °C. Up to 400 μL PI was added for staining and blending at 4 °C without light for 30 min. Testing was conducted through a computer, and the red fluorescence was recorded at an excitation wavelength of 488 nm.

#### 3.2.6. JC-1 Staining Method to Detect Mitochondrial Membrane Potential

The cells were dissociated and inoculated at the logarithmic phase into a six-well plate. The next day, the cells were added into the corresponding medicated medium according to the group setting. The negative control group was established. The cells were washed with PBS (centrifuged at 2000 rpm for 5 min) to collect and adjust the cell concentration to 1 × 10^6^/mL. Up to 100 μL of 10× incubation buffer was taken and diluted in 1× incubation buffer by 900 μL sterilized deionized water. The mixture was blended and preheated to 37 °C. We took 500 μL of 1× incubation buffer and added 1 μL JC-1 to prepare a JC-1 working solution by vortex blending. A total of 500 μL JC-1 working solution was taken to evenly suspend the cells, which were incubated for 15 min to 20 min in 5% CO_2_ at 37 °C. The solution was centrifuged at room temperature (2,000 rpm for 5 min) to collect cells, after which it was washed by 1× incubation buffer twice. The 500 μL of 1× incubation buffer was used to re-suspend the cells. Testing was conducted via a computer. The red fluorescence was recorded at an excitation wavelength of 488 nm. These mitochondrial membrane experiments have been observed by treatment of cells with FCCP, which evaluated our results.

#### 3.2.7. Western Blot Method to Detect Protein Expression

The cells were dissociated and inoculated at the logarithmic phase into a six-well plate. The next day, after the cells were adherent to the wall, they were added into the corresponding medicated medium according to the group setting. The negative control group was established. After 48 h of reaction, the cells were dissociated and collected with 0.25% trypsin for detection. A 70 μg protein sample was diluted to the same concentration with a pyrolysis buffer. An equal amount of sample loading buffer was placed in a test tube and cooled on ice after heating for 5 min at 95 °C to 100 °C for SDS-PAGE electrophoresis, which was conducted under 60 V constant voltage in the spacer gel for about 20 min and 80 V in the separation gel for about 80 min. After the electrophoresis, the protein was transferred to the PVDF membrane, which was sealed in 5% skimmed milk powder-confining liquid for 1 h at room temperature. The confining liquid was not washed. The confining liquid and the proper amount of primary antibodies (1:400) were added at about 0.1 mL/cm^2^. The solution was placed in the shaker and incubated at 4 °C overnight. The solution was then rinsed with PBST and filtered four times for 10 min each time. The secondary antibody with membrane and HRP (horseradish peroxidase-labeled antibody, secondary antibody diluted with confining fluid at 1:5,000) was shaken and incubated at room temperature for 1 h. The membrane was then fully washed with PBST and rinsed four times for 10 min each time. The dosage was calculated at 0.1 mL/cm^2^ developing liquid, which was added to the PVDF membrane and placed at room temperature for 1 min. The membrane was wrapped in a plastic film to avoid air bubbles as much as possible. The membrane protein was quickly wrapped on the X-ray film for exposure in the dark room. A processing machine was used to develop the images and photos. Finally, the exposure time was adjusted until the best band appeared.

### 3.3. Statistical Analyses

The experiment results were processed by SPSS software. They were denoted as *x* ± *s*, and statistically analyzed by the t test method (two-tailed).

## 4. Conclusions

This study found that GRg6 exhibits obvious anti-proliferative and apoptosis-inducing effects when it is applied to JK cells *in vitro*. GRg6 blocks *S* arrest in the cell cycle. The possible mechanisms of its effect could be related to the declining intracellular mitochondrial membrane potential and regulation of Bax and Bcl-2 protein expression in the Bcl-2 protein family of the mitochondrial membrane. This study provided an experimental basis for further development and utilization of active constituents in notoginseng.
